# Sleep Characteristics and Prevalence of Perceived Insufficient Sleep Across Age Groups in the Japanese Community-Based General Population: The Japan Multi-Institutional Collaborative Cohort Daiko Study

**DOI:** 10.3390/ijerph22091338

**Published:** 2025-08-27

**Authors:** Emi Morita, Hiroshi Kadotani, Naoto Yamada, Yoko Mitsuda, Takashi Tamura, Kenji Wakai

**Affiliations:** 1 International Institute for Integrative Sleep Medicine (WPI-IIIS), University of Tsukuba, 1-1-1 Tennodai, Tsukuba 305-8577, Ibaraki, Japan; 2 Forestry and Forest Products Research Institute, Forest Research and Management Organization, 1 Matsunosato, Tsukuba 305-8687, Ibaraki, Japan; 3 Department of Psychiatry, Shiga University of Medical Science, Seta Tsukinowa-cho, Otsu 520-2192, Shiga, Japan; kadotani@belle.shiga-med.ac.jp (H.K.); n-yamada@anzu.or.jp (N.Y.); 4 Kanbayashi Memorial Hospital, 89-1 Orikuchinishi, Oku-cho, Ichinomiya 491-0201, Aichi, Japan; 5 Department of Preventive Medicine, Nagoya University Graduate School of Medicine, 65 Tsurumai-cho, Showa-ku, Nagoya 466-8550, Aichi, Japanttamura@med.nagoya-u.ac.jp (T.T.); wakai.kenji.y2@f.mail.nagoya-u.ac.jp (K.W.)

**Keywords:** sleep duration, general population, actigraphy, sleep deprivation, individual differences, prevalence, chronotype

## Abstract

This study aimed to provide basic data according to age on objective sleep duration distribution and sleep characteristics via subjectivity, and to determine the prevalence of insufficient sleep and related sleep parameters in the general Japanese population. Data from the second survey of the Japan Multi-Institutional Cohort (J-MICC) Daiko Study were used for the analysis, with 2091 participants (1556 women; 58.6 ± 9.8 years old) included. Questionnaires included subjective sleep duration, perceived sufficiency, regularity, the Pittsburgh Sleep Quality Index (PSQI), Insomnia Severity Index (ISI), Morningness–Eveningness Questionnaire (MEQ), and Epworth Sleepiness Scale (ESS). Sleep measurements were taken via actigraphy for one week. In total, 247 (11.8%) respondents reported insufficient sleep and 953 (45.6%) reported somewhat insufficient sleep. Working-age adults had shorter subjective and measured sleep durations than those aged ≥ 60 years. About 20% of those aged ≥ 50 years and more than 30% of those aged < 50 years reported ≥2 h of sleep deprivation. Perceived insufficient sleep was associated with irregular sleep but not sleep efficiency or sleep latency. Additionally, sleep duration perceived as insufficient varied among individuals. Individual differences in sleep duration and sleep efficiency were greater than those based on age. In conclusion, implementing measures to address sleep deprivation in Japan’s working-age population are essential, and future epidemiological studies should consider individual differences.

## 1. Introduction

Statistical data from the Organization for Economic Cooperation and Development (OECD) reveal that Japanese people have the shortest subjective sleep duration compared to other countries [1]. Recent sleep measurements using wearable devices have also shown that Japan has a shorter sleep duration than other countries [2]. In a 2008 nationwide survey, the prevalence of insomnia, as determined through interviews, was 12.2% in men and 14.6% in women [3]. This, sleep-related issues are prevalent in Japan, making the improvement of sleep an important public health concern in the country.

The Japanese Ministry of Health, Labour and Welfare has been implementing the third term of National Health Promotion Measures in Japan in the 21st Century, known as “the third term of Health Japan 21 (HJ21)” since the fiscal year 2024. In this third term, the goal of HJ21 is to increase the number of people achieving sufficient sleep [4]. Here, “adequate sleep” is defined as 6 h to less than 9 h, and 6 h to less than 8 h for those aged 60 years and over [4].

In the aforementioned nationwide survey conducted by the Ministry of Health, Labour and Welfare, data were collected through a questionnaire on three items: average sleep duration, poor sleep quality (including difficulties falling asleep, maintaining sleep, or waking up early in the morning), and factors that interfere with getting enough sleep [5]. Additionally, most large cohort epidemiological studies in Japan have included only a few limited sleep-related items in their questionnaires. This is because there are very few large cohort studies focusing on sleep. Consequently, even the findings on subjective sleep status in large populations remain limited. For example, while chronotypes have been reported to change with age [6], as far as we are aware, there are very few studies that have reported the basic chronotype distribution by age in Japanese populations.

The understanding of sleep through objective assessments is even more limited. Although measurement data on sleep with wearable devices have been reported in recent years [2], in Japan, only a small number of epidemiological studies involving various health status and health-related factors in the general population have evaluated sleep duration through measurement. To our knowledge, there are only a few cases of measurements in populations exceeding 1000 people; a study using Holter recorders with tri-axial accelerometers and a few cohort studies in a local population are examples [7,8,9]. Since sleep duration and sleep characteristics differ among target populations, it is necessary to further accumulate objective sleep duration data for the Japanese population.

Furthermore, there are limited epidemiologic studies involving more than 1000 individuals that include sleep measurements, subjective sleep evaluations (such as daytime sleepiness, sleep quality), and health examination data [8]. Even among these research projects, there are few studies that comprehensively report age-specific data on measured sleep, insufficient sleep, chronotype, daytime sleepiness, subjective sleep quality, and insomnia symptoms in populations exceeding 1000 individuals.

Insufficient sleep syndrome is a key health issue in Japan, as highlighted by the OECD survey, which shows that sleep duration in Japan is the shortest of all member countries. Sleep duration is known to vary with age, so it is crucial to examine sleep duration by age. A meta-analysis of sleep duration assessed by polysomnography (PSG) across worldwide samples reports that, with increasing age, sleep durations for both total sleep time (TST) and time in bed (TIB) decrease, wake after sleep onset (WASO) increases, and sleep efficiency decreases [10]. However, Japanese epidemiological studies have also shown that older adults sleep longer both subjectively and objectively [7,11]. This discrepancy may be due to working-age people not getting enough sleep, resulting in a difference between the amount of sleep in daily life and the amount of sleep physiologically required. Therefore, understanding the prevalence of insufficient sleep by age and the association between insufficient sleep and measured sleep duration is necessary but remains unclear.

Although the definition of insufficient sleep varies across studies in the epidemiological research (i.e., short sleep duration, difference of sleep duration from sleep need, or perceived insufficient sleep), it is a common condition in populations [12,13]. The International Classification of Sleep Disorders (ICSD) 3rd edition mentions that the characteristics of insufficient sleep syndrome are high sleep efficiency over 90% and short sleep latency [14]. However, studies regarding the characteristics of objective sleep parameters for insufficient sleep syndrome are still limited. In particular, few studies have examined the characteristics of sleep latency and sleep efficiency in insufficient sleep among the general population in Japan.

As mentioned above, the current knowledge on the characteristics of sleep in the general population in Japan is insufficient, particularly regarding its distribution by age group. To implement public health measures regarding sleep, it is necessary to clarify the actual state of sleep by accumulating both objective sleep data and subjective sleep characteristic data in the general population.

Therefore, the aims of this study were (1) to provide basic data on the distribution of sleep duration via actigraphy and sleep characteristics via subjective reports across age groups in the general Japanese population, and (2) to determine the prevalence of perceived insufficient sleep across age groups and indicate sleep parameters related to perceived insufficient sleep.

## 2. Materials and Methods

### 2.1. Data Collection

In this study, we used data from a sleep survey conducted during a secondary survey of the Daiko Study, a component of the Japan Multi-Institute Collaborative Cohort (J-MICC) Study.

The J-MICC Study is a long-term genomic cohort study, initiated in 2005, aimed at preventing cancer and lifestyle-related diseases [15]. Participating institutions in the J-MICC study follow a standardized common protocol; questionnaires, health examinations, and the collection of genetically testable samples are being conducted, while monitoring cancer incidence and mortality until 2025.

A baseline survey of the Daiko Study was conducted between 2008 and 2010. The detailed protocol of the Daiko Study has been reported previously [16]. The participants were Nagoya residents aged 35−69 years at the time of the baseline survey, and all participants were volunteers. Recruitment was conducted primarily through leaflets and personal referrals until the total number of participants exceeded around 5000, regardless of sex.

Approximately 5 years after the baseline survey, the secondary survey was conducted targeting participants who had participated in the baseline survey and were being followed up in the cohort study. The participants were asked to visit Daiko Medical Center between January and December 2014 and participate in the survey following the standard protocol of the J-MICC Study, which included a health checkup, blood sample collection, and a questionnaire survey. Participants who agreed to participate in the above survey were subsequently asked to participate in an additional sleep survey that included a questionnaire on sleep and sleep measurement. The survey at the center was conducted on 3 weekdays and Saturdays per week during the period. After that, participants who did not visit the center were asked to respond to a questionnaire sent by mail as the secondary survey, although this was outside the scope of this study.

This study was approved by the Ethics Committee of Nagoya University Graduate School of Medicine (approval number 2008-0618-4), the Ethics Committee of the Faculty of Medicine, University of Tsukuba (approval number 254), and the Ethics Committee of the Forestry and Forest Products Research Institute. Written informed consent was obtained from all participants involved in this study.

### 2.2. Participants

In this study, we analyzed participants who took part in a sleep survey conducted as part of a secondary survey and who met the criteria. Figure S1 shows the flowchart of participant selection for analysis. We invited 2810 participants, who agreed to participate in the secondary survey at the center, to participate in the sleep survey, which involved sleep measurement using actigraphy and the completion of a sleep-rated questionnaire. Of these, 2277 participants agreed to participate, and we obtained valid actigraphy data from 2240 participants.

In this study, a total of 2091 participants (535 men and 1556 women) with a mean age of 58.6 ± 9.8 years (range: 39–75 years; 60.5 ± 9.9 years for men and 57.9 ± 9.7 years for women) were included in the analysis. The inclusion criteria were participants who consented to the sleep study, provided valid sleep measurements for at least three nights, and adequately answered all sleep-related and lifestyle questions in the questionnaire.

The reason for the higher number of female participants is that the survey at the center was also conducted on weekdays. It was difficult for people who work on weekdays to participate in the survey on weekdays. In Japan, the percentage of employment for women, especially the percentage of regular employment, is lower than that for men; therefore, there were more female participants.

### 2.3. Sleep Measurement

The participants were asked to wear an actigraphy device (MTI-210, ACOS Co., Ltd., Nagano, Japan) on their waist for a week, except during bath time, and complete a sleep diary [17,18]. Devices equipped with built-in sensors equivalent to MTI-210 demonstrated a high agreement with PSG, and the standard way to use them is to wear them around the waist [17,18]. During the data analysis of the actigraphy results, bedtime and get-up time were determined based on self-reports in the sleep diary. Actigraphy data were analyzed using the dedicated analysis software, Sleep Sing Act ver1.0 (SSA, Kissei Comtec Company Inc., Matsumoto City, Japan), to estimate sleep duration, sleep efficiency, and sleep latency.

Ineligible sleep data were excluded from the analysis of the actigraphy data, including those judged from sleep diary records to be different from normal sleep (e.g., nighttime vehicle journeys, hospitalization, late-night Olympic or World Cup watching, family misfortune, ill health, or travel/business trips), no sleep data recorded by the actigraph (e.g., discontinuation of measurement or not wearing the device), significant discrepancies between the sleep diary report and actigraphy data (for example, when the activity level recorded by actigraphy during the period between bedtime and wake-up time reported in the sleep diary was at the same level as the activity level during the day), or situations where appropriate sleep time data could not be obtained due to the data processing characteristics of the software (each day cannot be properly divided when the main sleep period includes noon).

Sleep duration per day was defined as the sum of all recorded sleep durations, including naps, within a 24 h period. The SSA software defines the longest sleep period within 24 h (from noon to noon the next day) as the main sleep period. Naps were defined as the second or subsequent length of sleep in a day. In this study, sleep efficiency, sleep latency, and WASO were obtained only from the main sleep phase, not including naps. Average values of sleep duration, sleep efficiency, sleep latency, and WASO during the measurement period were used in the analysis. The mean TIB for the main sleep was calculated by dividing the mean TST in the main sleep by the mean sleep efficiency, as the SSA ver1.0 specification does not output this value.

Based on the above, three types of measured sleep durations were considered in this study: total sleep duration per day (including naps; hereafter referred to as total sleep duration per day by actigraphy), TST during the main sleep, and TIB for the main sleep.

Since SSA software does not output the average total time of naps per day, the difference between the total sleep duration per day and TST during the main sleep can be considered the average time of naps per day.

### 2.4. Questionnaires

The standard common protocol in the J-MICC group included three questions on sleep: subjective sleep duration (“How many hours do you usually sleep per day?”) [h], sleep regularity (“Are your bedtime and wake-up times regular?”; regular, not regular), and perceived sleep sufficiency (“Do you think you usually get enough sleep?”; sufficient, somewhat insufficient, insufficient, or I do not know).

In addition to the above, the sleep survey conducted during the secondary survey of Daiko Study included the following assessments: Pittsburgh Sleep Quality Index (PSQI) for sleep quality, Insomnia Severity Index (ISI) for insomnia symptoms, Morningness-Eveningness Questionnaire (MEQ) for chronotype, and Epworth Sleepiness Scale (ESS) for excessive daytime sleepiness [19,20,21,22,23,24,25,26,27]. The Japanese versions of these four questionnaires were used.

Participants were also asked about their use of sedative-hypnotic drugs at least once a week (“Do you regularly (once a week or more) take sleep medication or sleep-inducing medication?”), required sleep duration (“How many hours of sleep do you think is enough for you? This may differ from your actual sleep time”), frequency of drinking alcohol (“Overall, how often do you drink alcohol on average?”, “every day” to “none”), nightcap (“Do you usually drink alcohol within two hours before going to bed?” and “Times per week”), smoking status (“Do you smoke?”; never, former, current), and exercise habit on leisure time activity (“How much exercise do you do on holidays and when you have free time?”, frequency; “none” to “five times or more per week”, times per session; “less than 30 min” to “4 h or more”, intensity; week to heavy).

### 2.5. Statistical Analysis

Participants were divided into four age groups: ≤49, 50s, 60s, and ≥70 years. The working-age group was defined as those under 60 years of age in this study, since many companies or public institutions in Japan have a mandatory retirement age of between 60 and 65 years.

We defined participants with a PSQI score > 5.5 as having poor subjective sleep quality, those with an ISI score ≥ 10 as having suspected insomnia, and those with an ESS score ≥ 11 as having daytime sleepiness [21,22,28]. The deprived sleep time was calculated by subtracting the average self-rate sleep duration from the self-reported required sleep duration. Chronotypes assessed by the MEQ were aggregated from five groups into three groups: morning type, intermediate type, and evening type. When examining the association between perceived sleep sufficiency and other factors, participants who reported not knowing their sleep sufficiency (n = 98) were excluded.

Habitual drinking alcohol was defined as drinking at least once a week. Habitual nightcap was defined as alcohol drinking within two hours before going to bed once a week or more. Habitual exercise was defined as ≥30 min of leisure time activity at least once a week, regardless of exercise intensity [29].

Differences in continuous variables between two groups were analyzed using the Student’s *t*-test, while one-way analysis of variance (ANOVA) was used for comparisons among three or more groups. Differences in proportions were assessed using the chi-squared test. The test for difference in proportions of ordinal variables used the Mantel–Haenszel test for trends. Spearman’s correlation coefficient was used to analyze correlations.

Perceived insufficient sleep was defined as those who responded “insufficient” in the sleep sufficiency questionnaire. In the logistic regression analysis, the objective variable was insufficient sleep, and the explanatory variables were sex, age group, chronotype, sleep regularity, shift work and one of the following factors related to sleep: self-rated sleep duration, total sleep duration per day, TST during the main sleep, TIB for the main sleep, sleep efficiency, and sleep latency.

The significance level was set at 5%. IBM SPSS Statistics version 23 for Windows (IBM, Armonk, NY, USA) was used for statistical analyses.

## 3. Results

### 3.1. Characteristics of Participants

Table 1 presents the characteristics of participants by sex. The mean age was significantly higher among men (men, 60.5 ± 9.9 years; women, 57.9 ± 9.7 years; *p* < 0.001). A total of 84 participants (4.0%) were shift workers. The percentages of participants with a BMI ≥ 25.0 (*p* < 0.001), nightcap (*p* < 0.001), drinking alcohol (*p* < 0.001), smoking (*p* < 0.001), and exercise habits (*p* = 0.01) were significantly higher in men compared to women.

Table S1 provides these characteristics by age group. Among men, over 90% of those under the age of 60 years had a job, while 55.9% of those in their 60s and 26.4% of those in their 70s had a job. Regarding lifestyle habits, the older age group had lower percentages of habitual nightcaps and smoking, and higher percentages of exercise habits. For women, more than 75% of those under the age of 60 years were employed, compared to 36.7% of those in their 60s and 21.6% of those in their 70s. Regarding lifestyle habits, the older age group had lower percentages of nightcap, drinking alcohol, and smoking, and higher percentages of exercise habits.

### 3.2. Subjective and Measured Sleep Duration, Sleep Efficiency, and Sleep Latency

The mean subjective sleep duration was 6.5 ± 1.0 h (range: 3.0–10.0 h). The mean total sleep time per day (TST during main sleep and naps) measured by actigraphy was 355.4 ± 59.6 min (range: 116.3–573.1 min). The TST during main sleep was 342.3 ± 62.2 min (range: 100.9–556.9 min) and TIB for the main sleep was 417.6 ± 62.6 min (range: 197.5–667.8 min). The mean sleep efficiency was 82.1 ± 9.9% (range: 27.1–97.5%).

Regarding subjective sleep duration, 498 people (23.8%) did not meet the criteria for adequate sleep duration by the Ministry of Health, Labour and Welfare [4]; short sleep duration: n = 347 (16.6%), long sleep duration: n = 151 (7.2%) (<60 years old: n = 6, 60≤ years old: n = 145). Regarding total sleep time (TST) per day, 1079 people (51.6%) did not meet the criteria; short sleep: n = 1056 (50.5%), long sleep: n = 23 (1.1%) (<60 years old: n = 1, 60≤ years old: n = 22).

The correlation coefficient between subjective sleep duration and total sleep duration per day was 0.38 (scatter plot in Figure S2). Total sleep duration, which excludes WASO, demonstrated a weak correlation as WASO is often imperceptible to individuals. In contrast, regarding TIB for the main sleep, the correlation coefficient was 0.54 (scatter plot in Figure S3). TIB, a measure perceivable by individuals, showed a moderate correlation with subjective sleep duration.

Table 2 summarizes subjective sleep duration and sleep outcomes measured by actigraphy across sex and age groups. Regarding sex differences, the total results show that men reported significantly longer subjective sleep durations (men, 6.6 ± 1.0 h; women, 6.4 ± 0.9 h; *p* < 0.001) and TIB for the main sleep (men, 426.2 ± 64.5 min; women, 414.7 ± 61.7 min; *p* < 0.001). However, among working-age participants, there were few differences between men and women in these two indicators (self-rated sleep duration, around 6.3 h; TIB, around 403 min in both men and women).

Men had significantly shorter total sleep durations per day (men, 343.4 ± 68.0 min; women, 359.5 ± 55.9 min; *p* < 0.001) and TST during the main sleep (men, 330.2 ± 68.2 min; women, 346.4 ± 59.5 min; *p* < 0.001) compared to women. Specifically, among working-age participants, men had total sleep durations per day and TST during the main sleep that were over 20 min shorter than those of women. A total of men also exhibited significantly lower sleep efficiency (men, 77.6 ± 11.5%; women 83.7 ± 8.7%; *p* < 0.001), longer WASO (men, 67.8 ± 41.2 min; women, 45.0 ± 32.8 min; *p* < 0.001), and longer sleep latency (men 19.0 ± 15.2 min; women 15.5 ± 10.3 min; *p* < 0.001), these indicating that men have a poorer sleep status compared to women.

In terms of age groups, subjective sleep duration, as well as the three measured sleep durations (total sleep duration per day, TIB for the main sleep, and TST during the main sleep) showed significant differences between age groups for both men and women (all *p* < 0.001). These sleep durations, both subjective and measured, were shorter for the working-age group than for those aged 60 years or older.

For sleep efficiency, there was no significant difference across age groups among men (*p* = 0.33). Although there were significant differences across age groups among women (*p* = 0.001), the mean difference was only 2% at most (highest in the 50s, 84.6 ± 8.0%; lowest in the 70s and older, 82.6 ± 9.2%). In total and in women, differences were more clearly visible in the distribution by age when sleep efficiency was divided into five groups (Figure S4A–C; total, *p* < 0.001; women, *p* < 0.001). The group over 60 years of age in women had a lower percentage of high sleep efficiency of 90% or more, at least seven percentage points lower than the working-age group. However, as Figure S4 shows, for both men and women, individual differences in sleep efficiency are greater than the differences between age groups. In each age group, sleep efficiency was widely distributed, ranging from less than 60% to 90% or over, indicating large individual differences.

On the other hand, sleep latency did not differ significantly between age groups for either sex (*p* = 0.84 for men and *p* = 0.39 for women).

### 3.3. Subjective Assessment of Sleep Characteristics

The characteristics of sleep based on subjective assessments by age and sex are presented in Table 3. The percentages of participants with subjective poor sleep quality (PSQI > 5.5) were 43.2%, insomnia symptoms (ISI ≥ 10) 31.1%, use of sedative–hypnotic drugs at least once a week 7.1%, and daytime sleepiness (ESS ≥ 11) 31.4%.

Among respondents, 86.8% reported having regular sleep patterns. Regarding the association between shift work and irregular sleep, 34.5% of shift workers (29 out of 84) and 12.3% of non-shift workers (247 out of 2007) reported irregular sleep patterns, with a significantly higher percentage of irregular sleepers among shift workers (*p* < 0.001). However, 65.5% of shift workers reported having regular sleep patterns, indicating that shift work and irregular sleep are not synonymous.

There were no significant differences between men and women for poor sleep quality (men, 41.9%; women, 43.7%; *p* = 0.46), insomnia symptoms (men, 31.2%, women, 31.0%; *p* = 0.94), use of sleeping medications (men, 7.5%; women, 7.0%; *p* = 0.71), daytime sleepiness (men, 32.3%; women, 31.0%; *p* = 0.58), regular sleep patterns (men, 85.6%; women, 87.2%; *p* = 0.34), or chronotype (*p* = 0.27).

Age-related differences showed that both men and women in older age groups had exhibited higher percentages of using sleeping pills and regular sleep, while those in the working-age group had exhibited a higher percentage of daytime sleepiness. Regarding chronotype, the younger group exhibited a higher percentage of evening types and a lower percentage of morning types, compared to the older group, regardless of sex.

Among men, a higher percentage of poor sleep quality was found in working-age groups. For women, there were no significant differences in poor sleep quality between age groups, but significant differences were found for insomnia symptoms, with higher percentage for those aged 70 years and older.

### 3.4. Perceived Insufficient Sleep

Regarding perceived sleep sufficiency, 793 participants (37.9%) reported sufficient sleep, 953 (45.6%) reported somewhat insufficient sleep, 247 (11.8%) reported insufficient sleep, and 98 (4.7%) reported “do not know.”

The prevalence of perceived insufficient sleep and the self-rated deprived sleep duration across sex and age groups is shown in Table 4. Women reported higher percentages of insufficient sleep (women, 12.9%, men 8.6%; *p* < 0.001) and self-rated sleep deprivation of more than 2 h (women 19.8%, men, 14.4%; *p* = 0.005), as well as longer deprived sleep duration (women, 0.9 ± 0.9 h; men, 0.7 ± 0.9 h; *p* < 0.001) compared to men.

Considering the age groups, both men and women in the working-age group were more than twice as likely to report “insufficient sleep” compared to those aged 60 years and older (working-age group 12.4% to 18.4%, while 3.2% to 8.6% in ≥60 years old). The required sleep duration was significantly different by age groups in women (*p* < 0.001); longer in the young age groups. Deprived sleep time varied by age, with both men and women in each age group under 60 years reporting sleep deprivation of at least 0.9 h on average. The proportion of individuals reporting sleep deprivation of ≥2 h was higher in the younger age groups in both sexes, with more than 30% of those under 50 years old and approximately 20% of those in their 50s.

The associations between perceived sleep sufficiency, participant characteristics, and sleep indicators are shown in Table 5. Insufficient sleep was significantly associated with women (*p* < 0.001), younger age (*p* < 0.001), evening chronotype (*p* < 0.001), irregular sleep patterns (*p* < 0.001), and shift work (*p* = 0.02). The insufficient sleep group had a significantly shorter subjective sleep duration, total sleep duration per day according to actigraphy, TST during main sleep, and TIB for the main sleep (all *p* < 0.001).

The difference in the total daily sleep time and TST during this sleep was greater in the insufficient sleep group (342.6 ± 64.7 min and 322.7 ± 68.3 min; approximately 20 min) than in the sufficient sleep group (370.6 ± 57.7 min and 359.7 ± 58.8 min; approximately 11 min). This indicates that the insufficient sleep group had a longer nap time. This may be related to the fact that shift workers who experience sleep fragmentation are more likely to be found in groups with sleep deprivation.

**Figure 1:**
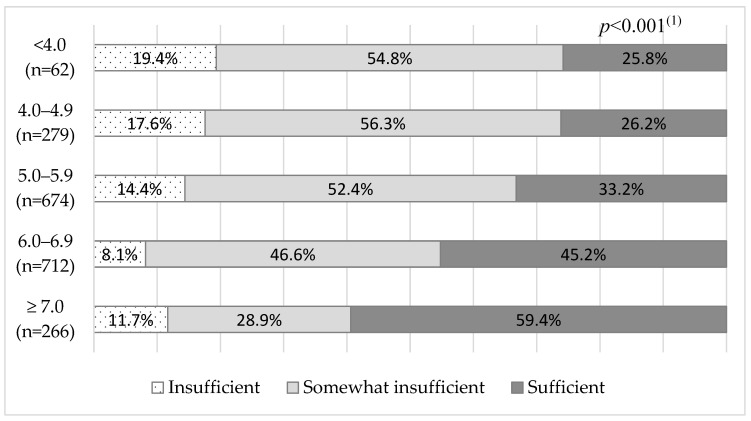
Total sleep duration per day by actigraphy and perceived sleep insufficiency. ^(1)^ Mantel–Haenszel test for trend.

Figure 1 illustrates the association between total sleep duration per day across the five age groups and perceived sleep sufficiency. Short total sleep duration per day groups indicated higher percentages of perceived insufficient sleep, while longer total sleep duration per day indicated a higher percentage of perceived sufficient sleep (*p* < 0.001). However, there were notable individual differences. Among participants with <4 h of total sleep duration per day, 25.8% reported “sufficient sleep”; conversely, 11.7% of those with ≥7 h of total sleep reported “insufficient sleep”.

Since the correlation between subjective and objective sleep duration was not necessarily high, we further examined the association between subjective sleep duration and perceived sleep sufficiency (Figure S5). Subjective sleep duration was inversely significantly associated with the percentage of perceived sleep insufficiency (*p* < 0.001), as was total sleep duration per day, though individual differences were observed.

As shown in Table 5, sleep efficiency tended to be slightly higher in the group that was not getting enough sleep (81.7% ± 10.0% for the sufficient sleep group, 82.3% ± 9.5% for the somewhat insufficient group, and 83.3% ± 9.5% for the insufficient group), but this was not statistically significant (*p* = 0.07). The sleep latency was 16.9 ± 13.1 min in the sufficient group, 15.8 ± 10.5 min in the somewhat insufficient group, and 16.3 ± 12.0 min in the insufficient group, with no significant difference (*p* = 0.20). However, sleep efficiency was significantly higher, and sleep latency was significantly shorter when subjective sleep duration or TIB during the main sleep was shorter (Table S2). In particular, sleep efficiency was lower (76.3 ± 10.1%), and sleep latency (27.8 ± 20.8 min) was longer in the group with a TIB ≥ 9 h.

The results of the logistic regression analysis are shown in Table S3. Perceived insufficient sleep, adjusted for sex, age group, chronotype, irregular sleep, and shift work, was significantly associated with subjective sleep duration (adjusted odds ratio [aOR]: 0.27, 95% confidence interval [CI]: 0.22–0.32), total sleep duration per day (aOR: 0.86, 95% CI: 0.75–0.99), TST during main sleep (aOR: 0.81, 95% CI: 0.70–0.93), and TIB for main sleep (aOR: 0.73, 95% CI: 0.64–0.84). The results show that insufficient sleep shows the highest association with subjective sleep duration. Among measured sleep durations, perceived insufficient sleep was more related to TIB than to TST, because TIB had a lower aOR compared to TST. Sleep efficiency and sleep latency were not significantly associated with perceived insufficient sleep after adjusting for sex, age, chronotype, irregular sleep, and shift work.

These logistic regression analyses indicated that insufficient sleep was significantly associated with sex, age, and irregular sleep patterns. The aOR for irregular sleep was 2.63 (95%CI: 1.82–3.80) when subjective sleep duration was included as an explanatory variable (Table S3: Model 1), and it was the largest when total sleep duration per day among four sleep durations was included (Table S3: Model 2; aOR: 4.70, 95%CI: 3.38–6.54).

Regarding chronotype and shift work, a significant association with perceived insufficient sleep was demonstrated (Table 5). However, the significant associations between perceived insufficient sleep and these two factors were no longer significant after adjusting for other sleep-related factors in the logistic regression analysis (Model 1 to Model 4 in Table S3).

## 4. Discussion

### 4.1. The Association Between Subjective and Objective Sleep

Previous population-based studies have reported that subjective and objective sleep duration differ, and their correlation has been characterized as weak to moderate [30,31]. This study’s results also support those of previous studies. Subjective sleep duration showed a weak correlation with TST (Spearman’s correlation coefficient = 0.38, Figure S2). The reason for the weak correlation with TST is thought to be that TST is affected by WASO, which is difficult to perceive.

On the other hand, since TIB is based on sleep diary records, it can be assumed that TIB and subjective sleep duration are almost identical in the absence of insomnia. However, the correlation between TIB and subjective sleep duration was only moderate (Spearman’s correlation coefficient = 0.54, Figure S3). The results of this study suggest that it may not be possible to recognize even subjective sleep duration without daily records.

Therefore, subjective sleep duration cannot substitute for objectively measured TST, and to determine objective sleep duration, it is necessary to measure it using a device.

### 4.2. Age and Sex Differences in Sleep Duration

Regarding sleep duration by age, a meta-analysis of sleep duration assessed by PSG globally shows that sleep duration shortens with age [10]. However, in this study, both subjective and objective sleep durations were shorter in the working-age group under 60 years and longer in the older age group (Table 2). The results are similar to those of previous large-scale studies on subjective or measured sleep duration in Japan [7,11]. This discrepancy in the trend of sleep duration by age group between PSG studies and studies in Japan may be attributed to differences in the conditions under which sleep was measured. The PSG assessments were conducted in laboratory settings without social time constraints, whereas our study was conducted in real-life conditions with such constraints.

Sleep duration is often limited among Japan’s working-age population due to work, child-rearing, and other social roles. The standard working hours for full-time workers in Japan are 40 h/week. Overtime is also common, resulting in an annual working time of approximately 2000 h [32]. In 2010, 32.0% of male workers and 11.1% of female workers worked 49 h or more per week, and in 2015, 29.5% of men and 9.5% of women did so [33]. Additionally, the average commute time in Japan is 1 h 19 min [34]. Siesta, which is an afternoon nap, is a practice observed in several dozen countries, primarily in tropical regions [35], and may be beneficial for compensating for short nighttime sleep. However, it does not exist in Japan. Furthermore, in the traditional way of thinking, it was considered a virtue to work long hours and sacrifice sleep. For these reasons, it is thought that the working generation in Japan has less time available for sleep.

On the other hand, many companies and public organizations in Japan set retirement ages between 60 and 65 years, and many employees in their 60s choose to reduce their working hours or retire completely. Therefore, it is thought that those aged 60 years and over have more time to sleep, as social factors that limit sleep time are no longer present.

### 4.3. Insufficient Sleep

According to an OECD survey, Japanese people have the shortest sleep duration [1]. We attribute this to sleep deprivation rather than biological factors. Only 37.9% of respondents in this study reported getting enough sleep (Table 4). Insufficient sleep is a common health issue among the Japanese population.

Furthermore, the percentage of people who reported insufficient sleep varied by age (Table 4). This study indicated that insufficient sleep is more severe among the working-age population. However, among the working-age population, there was little difference in subjective sleep duration and total daily sleep duration between those aged 49 years and under and those in their 50s (Table 2). Nevertheless, younger groups were more likely to feel sleep deprivation. The proportion of people who reported being deprived of 2 h or more of sleep was over 30% among those aged 49 years and under and approximately 20% among those in their 50s (Table 4). As indicated by a meta-analysis of PSG data, younger individuals physiologically require more sleep. Therefore, it is considered that younger age groups are more likely to feel sleep deprivation, even if they sleep the same amount as those in their 50s.

Next, we discuss daytime sleepiness. One of the diagnostic criteria for insufficient sleep syndrome is excessive daytime sleepiness [14]. Daytime sleepiness can affect work productivity and daily life. Therefore, it is important not to feel sleepy during the day. However, daytime sleepiness was also approximately 10 points higher among the working-age population than among those aged 60 years and older (Table 3). Additionally, among men of working age, a high proportion reported poor subjective sleep quality, and a low proportion had regular sleep patterns (Table 3). Working-age men were found to be at a higher risk with respect to sleep.

These results indicate that the issue of insufficient sleep in the working-age population requires urgent measures from a public health perspective.

### 4.4. Age and Sex Differences in Sleep Efficiency

Sleep efficiency differed between men and women, with men showing about 5% lower efficiency across all age groups (Table 2). This study did not investigate sleep apnea syndrome, which leads to decreased sleep efficiency, but it is possible that this is related to the high proportion of obesity (BMI ≥ 25.0; Table 1), which is a risk factor for sleep apnea syndrome, in men. Further data, including identifying sleep apnea, accumulation, and analysis are necessary.

Regarding sleep efficiency by age, the meta-analysis of sleep duration assessed by PSG shows that sleep efficiency decreases with age [10]. However, the mean sleep efficiency did not decrease by age among men and was slightly lower in the older age group among women in the present study (Table 2). Since the cause of these differences in results cannot be determined, further data accumulation and clarification in the general Japanese population are needed.

Individual differences in sleep efficiency were more pronounced than age-related differences (Figure S4). Low sleep efficiency is a known risk factor for lifestyle-related diseases [36]. Since sleep efficiency cannot be perceived without measurement, it is essential to identify high-risk groups through objective sleep measurements.

### 4.5. Use of Sleep Medication and Chronotype

This study provided data for the distribution of sedative–hypnotic drug use and chronotypes by age and sex in the Japanese general population (Table 3). In the general population, the proportion of sleeping pills taken varies between countries. In a Brazilian national survey of people over 18 years of age (mean age: 42.9 years), the percentage of use was 7.6% [37]. In the United States, the National Health Interview Survey found that the percentage of use in the last 30 days, either every day or most days, was 8.4% among adults aged 18 years or older [38]. A cohort study of more than 480,000 people aged ≥ 20 years (mean age: 40.3 ± 13.5 years) in Taiwan reported that the percentage of use was only 1.8% [39].

The percentage of use of sleeping pills at least once a week was 7.1% in the present study (Table 3). A study of approximately 4 years of medical records from nationwide Japanese health insurance members (range: 0–75 years old, mean age of 49.1 ± 15.1 years) found that 8.2% were prescribed sleeping pills at least once as outpatients during the 4-year period [40]. The results of this study support these findings, and although self-reported, we believe participants in this study responded appropriately. The higher age average (58.6 ± 9.8 years) in this study can contribute to a higher usage percentage; however, since the health insurance survey covered a 4-year period, while this study focused on a single point in time and due to the proportion of participants who reported weekly or more frequent use, the lower figures in this study are considered reasonable.

Studies of general populations in other countries have reported that the older the age and for female sex, the higher the proportion of sleep medication use [37,38,41]. The present study also partly supported these findings; the proportion of sleep medication use was higher in the older age group. The proportion of sleep medication use was not significantly different between sexes in total, but it was higher in women compared to men in the 70s age group.

Because of cultural and social differences among countries, the percentage of sleeping medication use, as well as the duration of sleep, varied from country to country. On the other hand, since the risk of sleep disorders is physiologically higher among the elderly, the percentage of taking sleeping medication increases among the elderly, a trend that is expected to be the same in different countries.

Regarding chronotypes, a study using the Munich ChronoType Questionnaire has reported that the morning type increases with age in adults [6]. The results of this study are similar to those of previous studies (Table 3). Regarding sex differences, the meta-analysis reported that males have a higher percentage of evening type [42]. However, this study found no significant difference between chronotype and sex.

### 4.6. Individual Differences in Sleep Duration

This study also highlighted individual differences in sleep duration. There was an inverse association between subjective and objective sleep durations and the perception of insufficient sleep (Table 5). However, individual differences were evident in what was considered sufficient. Some respondents reported that <4 h of total sleep duration per day was sufficient, while others reported that ≥7 h was insufficient (Figure 1). These findings suggest large individual differences in the amount of sleep perceived as insufficient.

Numerous epidemiological studies have reported that subjective sleep duration shows a U-shaped association with death and lifestyle-related diseases [43,44,45,46]. However, as shown in this study, there are individual differences in the amount of sleep perceived as sufficient or insufficient. Since optimal sleep duration varies among individuals, it may not be appropriate to uniformly recommend a sleep duration at the bottom of the U-shape observed in epidemiological studies. Most sleep epidemiology studies do not account for individual differences in required sleep duration or insufficient sleep. When examining the association between sleep duration and health in the future, these factors should be considered. In Japan, in particular, where many people suffer from sleep deprivation and a certain number spend their daily sleep time deviating from the required sleep duration, both sleep duration and insufficient sleep should be focused on when evaluating health and sleep duration.

Additionally, in many studies on short and long sleepers, as well as in genome-wide association studies, such as those mentioned below, the definition of short sleepers often refers to people whose sleep duration is short. However, we believe that it is necessary to distinguish between people who are forced to sleep for short periods of time as a result of sleep deprivation and those who, by nature, sleep for short periods of time.

Efforts to identify genes regulating sleep duration (i.e., individual differences in sleep duration) are ongoing. Genome-wide association studies have identified some candidate loci associated with a short or long sleep duration [47,48]. However, the mechanisms underlying individual differences remain unclear. Once clarified, personalized approaches to preventing sleep-related issues may become possible, highlighting the importance of further research in this area.

### 4.7. Characteristics of Perceived Insufficient Sleep

Short sleep latency and high sleep efficiency ≥ 90% have been cited as characteristics of insufficient sleep syndrome [14]. However, in this study, there were no significant differences in sleep latency or sleep efficiency between the group reporting perceived insufficient sleep and the group reporting perceived sufficient sleep (Table 5).

Sleep deprivation in interventional studies has been reported to increase sleep efficiency and to reduce sleep latency [49]. However, since sleep efficiency inherently varies among individuals, it may not be appropriate to uniformly list a sleep efficiency of 90% or higher as a characteristic of insufficient sleep syndrome. The same may apply to sleep latency.

The present study indicated that short sleep duration is not necessarily associated with perceived insufficient sleep (Figure 1). The definition of insufficient sleep may need to be discussed further in the future, considering whether it involves short sleep duration, perceived insufficient sleep, or both. It is also necessary to take individual differences into account.

Additionally, the present study showed that perceived insufficient sleep was strongly associated with irregular sleep (Table S3). Even after adjusting for sleep duration and lifestyle-related factors, irregular sleep had a large aOR (4.70, 95% CI 3.38–6.54 in the case of Model 2 in Table S3), indicating that it is a risk factor for insufficient sleep. On the other hand, shift work was not associated with insufficient sleep in logistic regression analysis (Table S3). Therefore, regular sleep patterns, regardless of whether one works shifts, were shown to be beneficial in preventing perceived insufficient sleep.

### 4.8. Limitations and Strengths of This Study and Future Aspects

This study has limitations. In this study, sleep was measured using actigraphy, which does not allow for the analysis of sleep structure. Future studies aiming to elucidate individual differences in sleep and the relationship between detailed sleep structure and health should include PSG-based assessments.

The strength of this study lies in its objective assessment of sleep characteristics across age groups in a general Japanese population of over 2000 individuals, utilizing a week-long actigraphy data. This study also noted individual differences in the amount of sleep needed. Since this is an area that remains poorly understood, this study provides new aspects in sleep epidemiology.

This study is an initial analysis of the association between perceived insufficient sleep and some sleep parameters, but in the future, we will conduct a detailed verifies of insufficient sleep and health or chronotype, as well as individual difference of sleep. Additionally, through this cohort study, we plan to examine the relationship between objective and subjective sleep and physical and mental health and, through follow-up studies, determine the impact of objective sleep duration and sleep efficiency on cancer incidence and all-cause mortality.

## 5. Conclusions

This study provided basic data on the distribution of sleep duration in both subjective and objective sleep duration, sleep efficiency, sleep latency, perceived insufficient sleep, daytime sleepiness, subjective sleep quality, insomnia symptoms, sleep medication use, and chronotypes by age and sex in the Japanese general population.

Since the correlation between subjective and objective sleep duration was weak, subjective sleep duration is not a substitute for objective sleep duration. Further accumulation of objective measurement data on sleep is necessary in epidemiological studies in Japan.

Regarding insufficient sleep, this study revealed that the working-age population had shorter sleep durations and a higher percentage of perceived insufficient sleep in Japan. Therefore, it is necessary to implement measures to ensure sufficient sleep for the working-age population. Notably, while insufficient sleep syndrome is believed to be characterized by high sleep efficiency and short sleep latency, this study found that perceived insufficient sleep was not associated with either high sleep efficiency or short sleep latency. The definition of insufficient sleep may need to be discussed further in the future, considering whether it involves short sleep duration, a subjective feeling of insufficient sleep, or both.

Additionally, the perception of sleep duration as insufficient varied among individuals. Individual differences in sleep duration and sleep efficiency were also greater than differences by age. Therefore, uniformly recommending a sleep duration at the bottom of the U-shape may not be appropriate. Individualized sleep measures are necessary for promoting health and preventing diseases, and future epidemiological studies should consider individual differences in characteristics, such as the required amount of sleep.

## Figures and Tables

**Table 1:** Characteristics of participants.

	Total (n = 2091)		Men (n = 535)		Women (n = 1556)		*p*-Value
	n	(%)	n	(%)	n	(%)	
Age [years] ^(1)^	58.6 ± 9.8		60.5 ± 9.9		57.9 ± 9.7		<0.001 ^(5)^
BMI ^(2)^ ≥ 25.0	289	(13.8)	130	(24.3)	159	(10.2)	<0.001 ^(6)^
Drinking alcohol ^(3)^	832	(39.8)	339	(63.4)	493	(31.7)	<0.001 ^(6)^
Nightcap ^(3)^	337	(16.1)	168	(31.4)	169	(10.9)	<0.001 ^(6)^
Current smoker	113	(5.4)	66	(12.3)	47	(3.0)	<0.001 ^(6)^
Exercise habits ^(4)^	1270	(60.7)	350	(65.4)	920	(59.1)	0.01 ^(6)^
People with jobs	1234	(59.0)	347	(64.9)	887	(57.0)	0.001 ^(6)^
Shift worker	84	(4.0)	21	(3.9)	63	(4.0)	0.90 ^(6)^

^(1)^ Mean ± standard deviation, ^(2)^ Boby Mass Index, ^(3)^ once a week or more, ^(4)^ at least once a week for 30 min or more on a leisure time activity, ^(5)^ Student’s *t*-test, and ^(6)^ chi-squared test.

**Table 2:** Measured sleep characteristics for sleep duration, efficiency, and latency by sex and age groups.

		Age Group (Years)	≤49	50s	60s	70≥	*p*-Value ^(1)^
Total		n = 2091	n = 467	n = 606	n = 648	n = 370	
	Self-rated sleep duration [h]	6.5 ± 1.0	6.3 ± 0.9	6.3 ± 0.9	6.6 ± 0.9	6.7 ± 1.0	<0.001
	Total sleep duration per day [min]	355.4 ± 59.6	349.5 ± 57.0	346.7 ± 56.0	358.9 ± 61.6	370.8 ± 61.8	<0.001
	Total sleep time during the main sleep [min]	342.3 ± 62.2	333.5 ± 59.8	332.9 ± 58.4	347.1 ± 64.1	360.3 ± 63.3	<0.001
	Time in bed for the main sleep [min]	417.6 ± 62.6	402.5 ± 63.4	402.0 ± 57.4	428.1 ± 59.5	444.2 ± 62.6	<0.001
	Wake after sleep onset [min]	50.9 ± 36.5	45.1 ± 34.1	45.6 ± 33.5	55.6 ± 37.7	58.6 ± 39.3	<0.001
	Sleep efficiency [%]	82.1 ± 9.9	83.1 ± 9.2	82.9 ± 9.5	81.2 ± 10.4	81.3 ± 10.1	0.001
	Sleep latency [min]	16.4 ±11.9	16.0 ± 12.5	15.9 ± 10.6	17.0 ± 12.1	16.5 ± 12.7	0.38
Men		n = 535	n = 97	n = 125	n = 188	n = 125	
	Self-rated sleep duration [h]	6.6 ± 1.0	6.3 ± 1.0	6.3 ± 0.9	6.7 ± 1.0	7.0 ± 0.9	<0.001
	Total sleep duration per day [min]	343.4 ± 68.0	328.6 ± 57.7	323.9 ± 63.4	346.8 ± 69.2	369.1 ± 69.6	<0.001
	Total sleep time during the main sleep [min]	330.2 ± 68.2	315.4 ± 56.0	309.8 ± 64.2	333.4 ± 69.9	357.5 ± 68.9	<0.001
	Time in bed for the main sleep [min]	426.2 ± 64.5	403.6 ± 59.6	405.9 ± 58.3	432.3 ± 62.5	454.6 ± 64.5	<0.001
	Wake after sleep onset [min]	67.8 ± 41.2	60.5 ± 36.0	67.2 ± 41.4	70.3 ± 42.4	70.3 ± 42.7	0.23
	Sleep efficiency [%]	77.6 ± 11.5	78.6 ± 10.4	76.4 ± 11.7	77.2 ± 12.0	78.7 ± 11.2	0.33
	Sleep latency [min]	19.0 ± 15.2	19.9 ± 19.9	19.0 ± 12.7	19.2 ± 14.2	18.1 ± 15.0	0.84
Women		n = 1556	n = 370	n = 481	n = 460	n = 245	
	Self-rated sleep duration [h]	6.4 ± 0.9	6.3 ± 0.9	6.3 ± 0.9	6.5 ± 0.9	6.5 ± 1.0	<0.001
	Total sleep duration per day [min]	359.5 ± 55.9	355.0 ± 55.6	352.7 ± 52.4	363.8 ± 57.6	371.7 ± 57.5	<0.001
	Total sleep time during the main sleep [min]	346.4 ± 59.5	338.2 ± 59.9	339.0 ± 55.3	352.8 ± 60.7	361.7 ± 60.3	<0.001
	Time in bed for the main sleep [min]	414.7 ± 61.7	402.2 ± 64.4	400.9 ± 57.1	426.3 ± 58.2	438.9 ± 61.1	<0.001
	Wake after sleep onset [min]	45.0 ± 32.8	41.1 ± 32.5	40.0 ± 28.7	49.5 ± 33.9	52.6 ± 36.2	<0.001
	Sleep efficiency [%]	83.7 ± 8.7	84.2 ± 8.5	84.6 ± 8.0	82.8 ± 9.2	82.6 ± 9.2	0.001
	Sleep latency [min]	15.5 ± 10.3	15.0 ± 9.4	15.1 ± 9.9	16.1 ± 11.0	15.7 ± 11.3	0.39

All values are mean ± standard deviation, ^(1)^ one-way ANOVA.

**Table 3:** Subjective assessment of sleep characteristics by sex and age.

Age Group		Total		≤49		50s		60s		70≥		*p*-Value ^(6)^
		n	(%)	n	(%)	n	(%)	n	(%)	n	(%)	
Total		n = 2091		n = 467		n = 606		n = 648		n = 370		
	PSQI ^(1)^ > 5.5	904	(43.2)	219	(46.9)	264	(43.6)	266	(41.0)	155	(41.9)	0.08
	ISI ^(2)^ ≥ 10	650	(31.1)	140	(30.0)	175	(28.9)	199	(30.7)	136	(36.8)	0.04
	Use of sedative–hypnotic drugs ^(3)^	149	(7.1)	17	(3.6)	25	(4.1)	48	(7.4)	59	(15.9)	<0.001
	ESS ^(4)^ ≥ 11	656	(31.4)	197	(42.2)	217	(35.8)	172	(26.5)	70	(18.9)	<0.001
	Regular sleep	1815	(86.8)	383	(82.0)	521	(86.0)	580	(89.5)	331	(89.5)	<0.001
	MEQ ^(5)^											
	Definitely morning type	90	(4.3)	13	(2.8)	11	(1.8)	43	(6.6)	23	(6.2)	<0.001
	Moderately morning type	762	(36.4)	121	(25.9)	205	(33.8)	247	(38.1)	189	(51.1)	
	Intermediate	1175	(56.2)	296	(63.4)	375	(61.9)	349	(53.9)	155	(41.9)	
	Moderately evening type	61	(2.9)	36	(7.7)	14	(2.3)	8	(1.2)	3	(0.8)	
	Definitely evening type	3	(0.1)	1	(0.2)	1	(0.2)	1	(0.2)	0	(0.0)	
Men		n = 535		n = 97		n = 125		n = 188		n = 125		
	PSQI ^(1)^ > 5.5	224	(41.9)	52	(53.6)	67	(53.6)	65	(34.6)	40	(32.0)	<0.001
	ISI ^(2)^ ≥ 10	167	(31.2)	30	(30.9)	48	(38.4)	49	(26.1)	40	(32.0)	0.501
	Use of sedative–hypnotic drugs ^(3)^	40	(7.5)	5	(5.2)	6	(4.8)	13	(6.9)	16	(12.8)	0.02
	ESS ^(4)^ ≥ 11	173	(32.3)	46	(47.4)	52	(41.6)	55	(29.3)	20	(16.0)	<0.001
	Regular sleep	458	(85.6)	74	(76.3)	101	(80.8)	170	(90.4)	113	(90.4)	<0.001
	MEQ ^(5)^											
	Definitely morning type	31	(5.8)	3	(3.1)	4	(3.2)	15	(8.0)	9	(7.2)	<0.001
	Moderately morning type	197	(36.8)	25	(25.8)	37	(29.6)	80	(42.6)	55	(44.0)	
	Intermediate	292	(54.6)	61	(62.9)	81	(64.8)	91	(48.4)	59	(47.2)	
	Moderately evening type	15	(2.8)	8	(8.2)	3	(2.4)	2	(1.1)	2	(1.6)	
	Definitely evening type	0	(0.0)	0	(0.0)	0	(0.0)	0	(0.0)	0	(0.0)	
Women		n = 1556		n = 370		n = 481		n = 460		n = 245		
	PSQI ^(1)^ > 5.5	680	(43.7)	167	(45.1)	197	(41.0)	201	(43.7)	115	(46.9)	0.60
	ISI ^(2)^ ≥ 10	483	(31.0)	110	(29.7)	127	(26.4)	150	(32.6)	96	(39.2)	0.005
	Use of sedative–hypnotic drugs ^(3)^	109	(7.0)	12	(3.2)	19	(4.0)	35	(7.6)	43	(17.6)	<0.001
	ESS ^(4)^ ≥ 11	483	(31.0)	151	(40.8)	165	(34.3)	117	(25.4)	50	(20.4)	<0.001
	Regular sleep	1357	(87.2)	309	(83.5)	420	(87.3)	410	(89.1)	218	(89.0)	0.02
	MEQ ^(5)^											
	Definitely morning type	59	(3.8)	10	(2.7)	7	(1.5)	28	(6.1)	14	(5.7)	<0.001
	Moderately morning type	565	(36.3)	96	(25.9)	168	(34.9)	167	(36.3)	134	(54.7)	
	Intermediate	883	(56.7)	235	(63.5)	294	(61.1)	258	(56.1)	96	(39.2)	
	Moderately evening type	46	(3.0)	28	(7.6)	11	(2.3)	6	(1.3)	1	(0.4)	
	Definitely evening type	3	(0.2)	1	(0.3)	1	(0.2)	1	(0.2)	0	(0.0)	

^(1)^ Pittsburgh Sleep Quality Index, ^(2)^ Insomnia Severity Index, ^(3)^ once a week or more, ^(4)^ Epworth Sleepiness Scale, ^(5)^ Morningness–Eveningness Questionnaire, and ^(6)^ Mantel–Haenszel test for trend.

**Table 4:** Self-rated assessment for perceived sleep insufficiency, sleep requirements, and sleep deprivation hours by sex and age.

	Age Group	Total		≤49		50s		60s		70≥		*p*-Value
		n	(%)	n	(%)	n	(%)	n	(%)	n	(%)	
Total		n = 2091		n = 467		n = 606		n = 648		n = 370		
	Perceived sleep sufficiency											<0.001 ^(2)^
	Sufficient	793	(37.9)	111	(23.8)	186	(30.7)	306	(47.2)	190	(51.4)	
	Somewhat insufficient	953	(45.6)	257	(55.0)	301	(49.7)	253	(39.0)	142	(38.4)	
	Insufficient	247	(11.8)	80	(17.1)	95	(15.7)	47	(7.3)	25	(6.8)	
	Don not know	98	(4.7)	19	(4.1)	24	(4.0)	42	(6.5)	13	(3.5)	
	Required sleep duration [h] ^(1)^	7.3 ± 0.8		7.5 ± 0.8		7.3 ± 0.8		7.2 ± 0.8		7.2 ± 0.8		<0.001 ^(3)^
	Lack of sleep duration [h] ^(1)^	0.8 ± 0.9		1.2 ± 1.0		1.0 ± 0.8		0.7 ± 0.8		0.5 ± 0.8		<0.001 ^(3)^
	Lacking 2 h or more of sleep	385	(18.4)	158	(33.8)	125	(20.6)	71	(11.0)	31	(8.4)	<0.001 ^(2)^
Men		n = 535		n = 97		n = 125		n = 188		n = 125		
	Perceived sleep sufficiency											<0.001 ^(2)^
	Sufficient	241	(45.0)	25	(25.8)	47	(37.6)	90	(47.9)	79	(63.2)	
	Somewhat insufficient	230	(43.0)	54	(55.7)	53	(42.4)	82	(43.6)	41	(32.8)	
	Insufficient	46	(8.6)	12	(12.4)	21	(16.8)	9	(4.8)	4	(3.2)	
	Don not know	18	(3.4)	6	(6.2)	4	(3.2)	7	(3.7)	1	(0.8)	
	Required sleep duration [h] ^(1)^	7.3 ± 0.8		7.5 ± 0.8		7.3 ± 0.9		7.3 ± 0.8		7.4 ± 0.7		0.20 ^(3)^
	Lack of sleep duration [h] ^(1)^	0.7 ± 0.9		1.2 ± 0.9		0.9 ± 0.9		0.6 ± 0.8		0.4 ± 0.8		<0.001 ^(3)^
	Lacking 2 h or more of sleep	77	(14.4)	31	(32.0)	25	(20.0)	16	(8.5)	5	(4.0)	<0.001 ^(2)^
Women		n = 1556		n = 370		n = 481		n = 460		n = 245		
	Perceived sleep sufficiency											<0.001 ^(2)^
	Sufficient	552	(35.5)	86	(23.2)	139	(28.9)	216	(47.0)	111	(45.3)	
	Somewhat insufficient	723	(46.5)	203	(54.9)	248	(51.6)	171	(37.2)	101	(41.2)	
	Insufficient	201	(12.9)	68	(18.4)	74	(15.4)	38	(8.3)	21	(8.6)	
	Don not know	80	(5.1)	13	(3.5)	20	(4.2)	35	(7.6)	12	(4.9)	
	Required sleep duration [h] ^(1)^	7.3 ± 0.8		7.5 ± 0.8		7.3 ± 0.8		7.2 ± 0.7		7.2 ± 0.9		<0.001 ^(3)^
	Lack of sleep duration [h] ^(1)^	0.9 ± 0.9		1.2 ± 1.0		1.0 ± 0.8		0.7 ± 0.8		0.6 ± 0.8		<0.001 ^(3)^
	Lacking 2 h or more of sleep	308	(19.8)	127	(34.3)	100	(20.8)	55	(12.0)	26	(10.6)	<0.001 ^(2)^

^(1)^ Mean ± standard deviation, ^(2)^ Mantel–Haenszel test for trend, ^(3)^ one-way ANOVA.

**Table 5:** Association between perceived sleep sufficiency and sleep characteristics.

Subjective Sleep Sufficiency	Insufficient		Somewhat Insufficient		Sufficient		*p*-Value
	n	(%)	n	(%)	n	(%)	
Sex							<0.001 ^(2)^
Men	46	(8.9)	230	(44.5)	241	(46.6)	
Women	201	(13.6)	723	(49.0)	552	(37.4)	
Age [years] ^(1)^	55.0 ± 9.3		57.0 ± 9.8		61.4 ± 9.4		<0.001 ^(3)^
Chronotype							<0.001 ^(2)^
Evening type	13	(21.0)	39	(62.9)	10	(16.1)	
Intermediate	160	(14.4)	567	(51.1)	383	(34.5)	
Morning type	74	(9.0)	347	(42.3)	400	(48.7)	
Regularity of sleep							<0.001 ^(2)^
Yes	160	(9.2)	822	(47.3)	757	(43.5)	
No	87	(34.3)	131	(51.6)	36	(14.2)	
Shift worker							0.02 ^(2)^
Yes	16	(20.3)	39	(49.4)	24	(30.4)	
No	231	(12.1)	914	(47.8)	769	(40.2)	
Self-rated sleep duration [h] ^(1)^	5.5 ± 0.9		6.2 ± 0.8		7.1 ± 0.8		<0.001 ^(3)^
Total sleep duration per day [min] ^(1)^	342.6 ± 64.7		345.5 ± 55.8		370.6 ± 57.7		<0.001 ^(3)^
Total sleep time during the main sleep [min] ^(1)^	322.7 ± 68.3		332.7 ± 58.8		359.7 ± 58.8		<0.001 ^(3)^
Time in bed for the main sleep [min] ^(1)^	388.0 ± 73.3		404.8 ± 59.6		441.0 ± 53.9		<0.001 ^(3)^
Sleep efficiency [%] ^(1)^	83.3 ± 9.5		82.3 ± 9.5		81.7 ± 10.0		0.07 ^(3)^
Sleep latency [min] ^(1)^	16.3 ± 12.0		15.8 ± 10.5		16.9 ± 13.1		0.20 ^(3)^

^(1)^ Mean ± standard deviation, ^(2)^ Mantel–Haenszel test for trend, ^(3)^ one-way ANOVA.

## Data Availability

Data is contained within the article and Supplementary Materials.

## Supplementary Materials

The following supporting information can be downloaded at: https://www.mdpi.com/article/10.3390/ijerph22091338/s1, Table S1: Characteristics of participants by sex and age; Table S2: Association of sleep efficiency and sleep latency with sleep duration; Table S3: Adjusted odds ratio for perceived insufficient sleep in logistic regression analysis; Figure S1: Flowchart of participant selection for analysis; Figure S2: Correlation between subjective sleep and measured total sleep duration per day by actigraphy; Figure S3: Correlation between subjective sleep duration and measured time in bed for the main sleep; Figure S4: Distribution of sleep efficiency by age groups; Figure S5: Self-rated sleep duration and perceived sleep insufficiency.

## Abbreviations

ESS, Epworth Sleepiness Scale; ISI, Insomnia Severity Index; MEQ, Morningness–Eveningness Questionnaire; OECD, Organization for Economic Cooperation and Development; PSG, polysomnography; PSQI, Pittsburgh Sleep Quality Index; TIB, time in bed; TST, total sleep time; aOR, adjusted Odds Ratio; CI, confidence interval.
