# Supplementary material for: Sleep Characteristics and Prevalence of Perceived Insufficient Sleep Across Age Groups in the Japanese Community-Based General Population: The Japan Multi-Institutional Collaborative Cohort Daiko Study
Source: Int J Environ Res Public Health. 2025 Aug 27;22(9):1338. doi: 10.3390/ijerph22091338 (PMC12469467; doi:10.3390/ijerph22091338)
Supplement: Supplementary file 1 [file ijerph-22-01338-s001.zip › ijerph-3525147-supplementary.pdf]

Table S1. Characteristics of participants by sex and age

**Age group [years]**	**≤49**		**50s**		**60s**		**≥70**		***p*-value[4)]**
	**n**	**(%)**	**n**	**(%)**	**n**	**(%)**	**n**	**(%)**	
Total	n=476		n=606		n=648		n=370		
BMI[1)]≥25.0	51	(10.9)	76	(12.5)	103	(15.9)	59	(15.9)	0.01
Drinking alcohol[2)]	185	(39.6)	241	(39.8)	267	(41.2)	139	(37.6)	0.78
Nightcap[2)]	100	(21.4)	98	(16.2)	95	(14.7)	44	(11.9)	<0.001
Current smoker	36	(7.7)	35	(5.8)	31	(4.8)	11	(3.0)	0.002
Exercise habits[3)]	200	(42.8)	319	(52.6)	459	(70.8)	292	(78.9)	<0.001
People with jobs	389	(83.3)	485	(80.0)	274	(42.3)	86	(23.2)	<0.001
Shift worker	24	(5.1)	37	(6.1)	12	(1.9)	11	(3.0)	0.003
Men	n=97		n=125		n=188		n=125		
BMI≥25.0	19	(19.6)	31	(24.8)	51	(27.1)	29	(23.2)	0.49
Drinking alcohol	54	(55.7)	74	(59.2)	130	(69.1)	81	(64.8)	0.06
Nightcap	39	(40.2)	40	(32.0)	57	(30.3)	32	(25.6)	0.02
Current smoker	17	(17.5)	22	(17.6)	20	(10.6)	7	(5.6)	0.001
Exercise habits	42	(43.3)	69	(55.2)	135	(71.8)	104	(83.2)	<0.001
People with jobs	89	(91.8)	120	(96.0)	105	(55.9)	33	(26.4)	<0.001
Shift worker	5	(5.2)	4	(3.2)	5	(2.7)	7	(5.6)	0.89
Women	n=370		n=481		n=460		n=245		
BMI≥25.0	32	(8.6)	45	(9.4)	52	(11.3)	30	(12.2)	0.09
Drinking alcohol	131	(35.4)	167	(34.7)	137	(29.8)	58	(23.7)	0.001
Nightcap	61	(16.5)	58	(12.1)	38	(8.3)	12	(4.9)	<0.001
Current smoker	19	(5.1)	13	(2.7)	11	(2.4)	4	(1.6)	0.01
Exercise habits	158	(42.7)	250	(52.0)	324	(70.4)	188	(76.7)	<0.001
People with jobs	300	(81.1)	365	(75.9)	169	(36.7)	53	(21.6)	<0.001
Shift worker	19	(5.1)	33	(6.9)	7	(1.5)	4	(1.6)	<0.001

[1)] Body Mass Index, [2)] Once a week or more, [3)] Leisure time activity (once a week for 30 minutes or more), [4)] Mantel–Haenszel test for trend

Table S2. Association of sleep efficiency and sleep latency with sleep duration

	n	Sleep Efficiency[1] [%]	*p*-value[2]	Sleep Latency [1] [min]	*p*-value[2]
Self-rated sleep duration			0.001		0.01
<5.0 [h]	50	82.9±10.5		18.0±14.9	
5.0 - 5.9 [h]	297	82.6± 9.5		15.6±10.8	
6.0 - 6.9 [h]	809	82.9± 9.4		15.4±10.3	
7.0 - 7.9 [h]	697	81.5±10.2		17.2±11.8	
8.0 ≤ [h]	238	80.5±10.5		17.9±16.6	
Time in Bed during the main sleep			<0.001		<0.001
<5.0 [h]	83	83.4± 8.7		14.0±9.4	
5.0 - 5.9 [h]	275	82.9±10.1		14.9±10.5	
6.0 - 6.9 [h]	696	82.7±10.0		15.6±10.6	
7.0 - 7.9 [h]	724	82.3± 9.5		16.2±11.0	
8.0 -8.9 [h]	267	79.9± 9.9		19.2±15.3	
9.0 ≤ [h]	46	76.3±10.1		27.8±20.8	

1) Mean ± standard deviation, 2) Linear regression model.

Table S3. Adjusted odds ratio for perceived insufficient sleep in logistic regression analysis

	Model 1		Model 2		Model 3		Model 4		Model 5		Model 6	
	aOR[1)]	95%CI[2)]	aOR	95%CI	aOR	95%CI	aOR	95%CI	aOR	95%CI	aOR	95%CI
Sex: Women (ref. Men)	1.41	(0.96-2.08)	1.66	(1.16-2.38)	1.69	(1.18-2.42)	1.53	(1.07-2.19)	1.51	(1.05-2.17)	1.59	(1.11-2.27)
Age group (years)												
≤49	1.99	(1.14-3.47)	2.31	(1.40-3.80)	2.23	(1.35-3.68)	2.03	(1.22-3.37)	2.37	(1.44-3.90)	2.40	(1.46-3.95)
50s	2.09	(1.22-3.56)	2.20	(1.35-3.56)	2.13	(1.31-3.46)	1.94	(1.19-3.17)	2.28	(1.41-3.69)	2.30	(1.42-3.73)
60s	1.07	(0.61-1.90)	1.06	(0.63-1.79)	1.05	(0.62-1.76)	1.03	(0.61-1.73)	1.09	(0.65-1.82)	1.08	(0.65-1.82)
70s	1	Ref	1	Ref	1	Ref	1	Ref	1	Ref	1	Ref
Chronotype												
Evening type	1.11	(0.51-2.43)	0.93	(0.45-1.92)	0.93	(0.45-1.92)	0.97	(0.47-2.01)	0.95	(0.46-1.96)	0.93	(0.45-1.93)
Intermediate	1.29	(0.92-1.81)	1.18	(0.86-1.61)	1.19	(0.87-1.62)	1.18	(0.86-1.62)	1.18	(0.86-1.61)	1.18	(0.86-1.61)
Morning type	1	Ref	1	Ref	1	Ref	1	Ref	1	Ref	1	Ref
Non-regularity of sleep	2.63	(1.82-3.80)	4.70	(3.38-6.54)	4.45	(3.19-6.21)	4.22	(3.01-5.90)	4.96	(3.57-6.89)	4.91	(3.53-6.82)
Shift work	1.09	(0.55-2.16)	1.06	(0.57-1.96)	1.05	(0.57-1.95)	1.04	(0.56-1.94)	1.03	(0.56-1.91)	1.03	(0.56-1.91)
Self-rated sleep duration	0.27	(0.22-0.32)										
Total sleep duration per day			0.86	(0.75-0.99)								
Total sleep time during the main sleep					0.81	(0.70-0.93)						
Time in Bed during the main sleep							0.73	(0.64-0.84)				
Sleep efficiency									1.01	(0.99-1.03)		
Sleep latency											1.00	(0.99-1.01)

1) adjusted Odds Ratio, 2) Confidence Interval

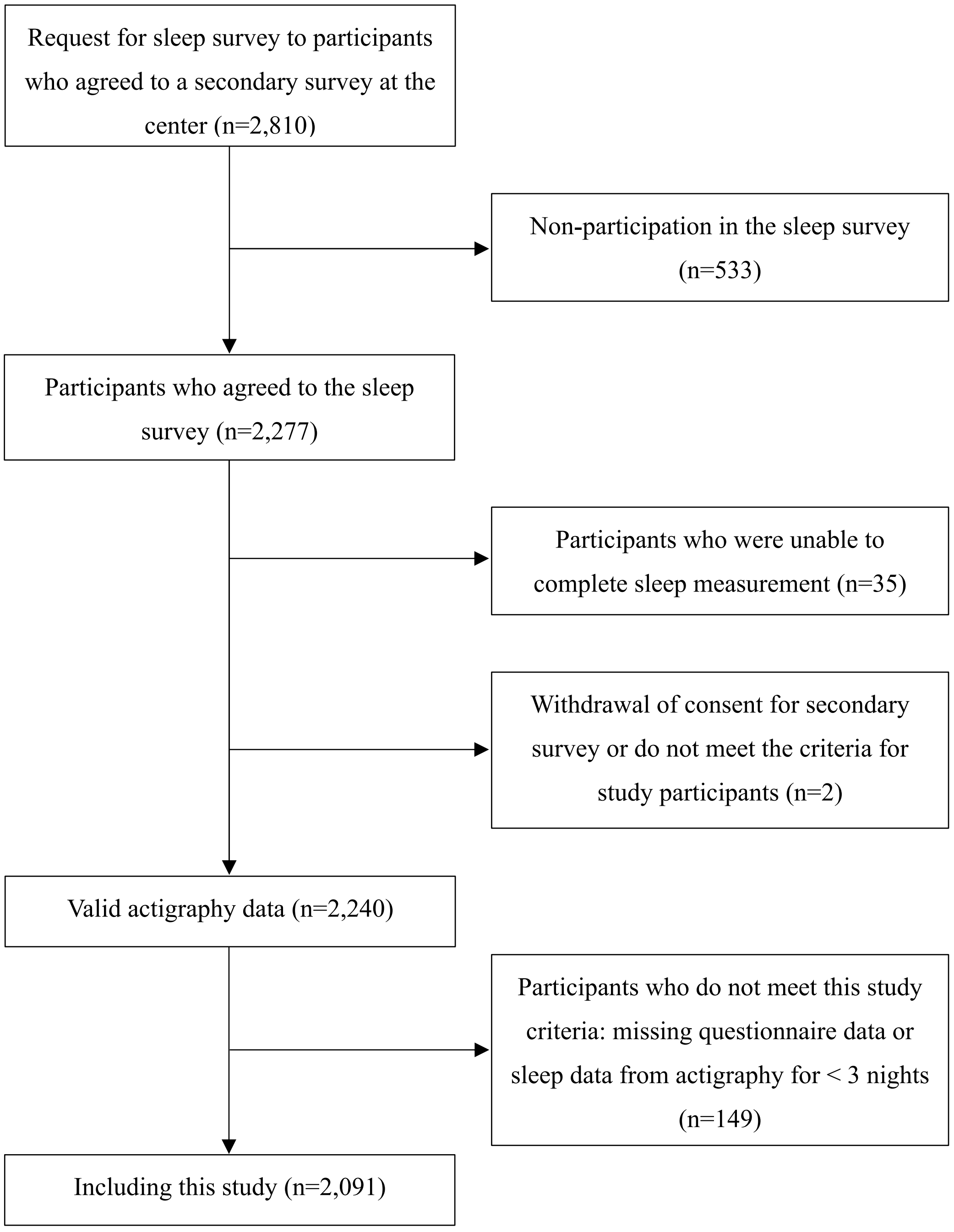

Figure S1. Flowchart of participant selection for analysis

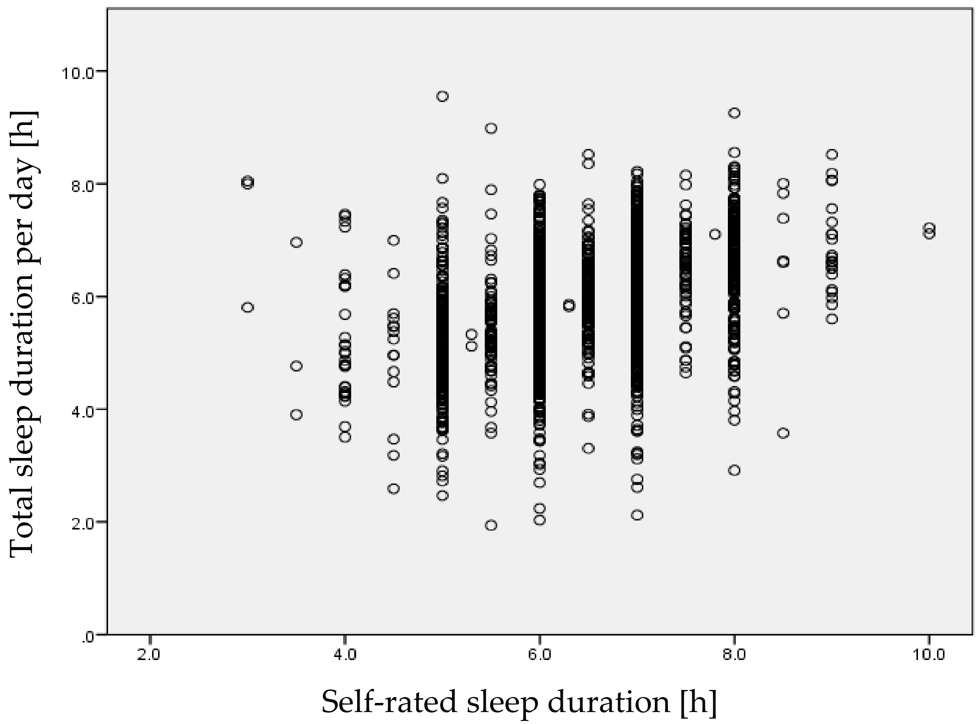

Figure S2. Correlation between subjective sleep and measured total sleep duration per day by actigraphy

Spearman's correlation coefficient = 0.38

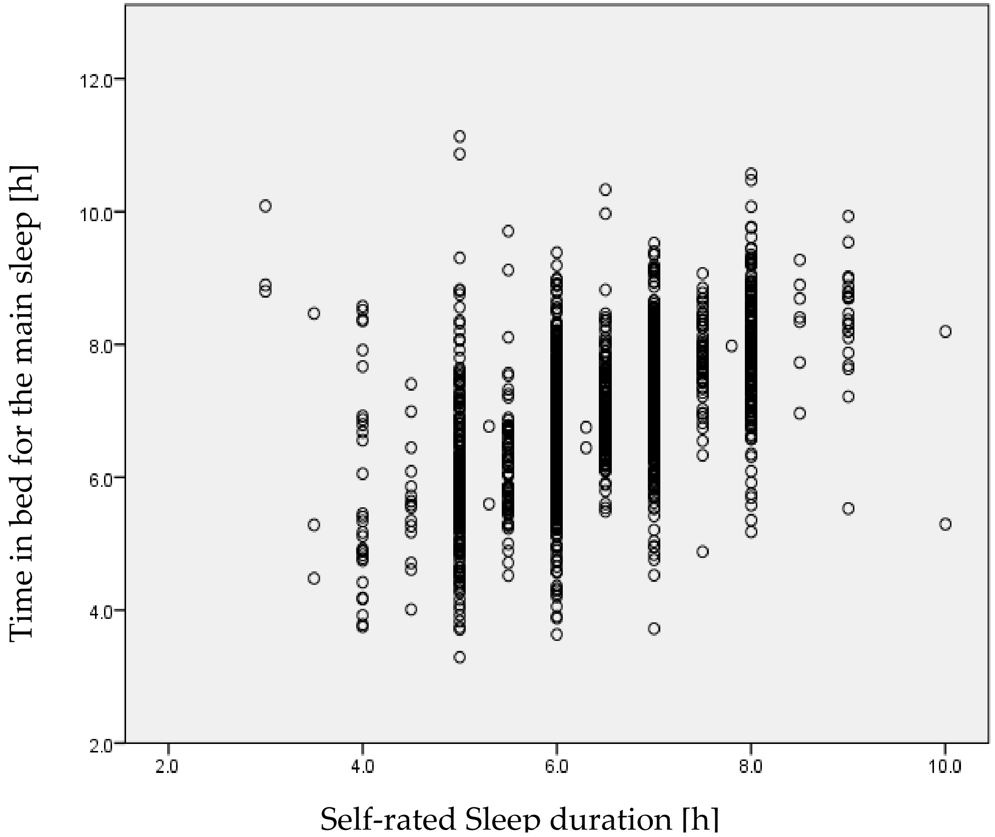

Figure S3. Correlation between subjective sleep duration and measured time in bed during the main sleep

Spearman's correlation coefficient = 0.54

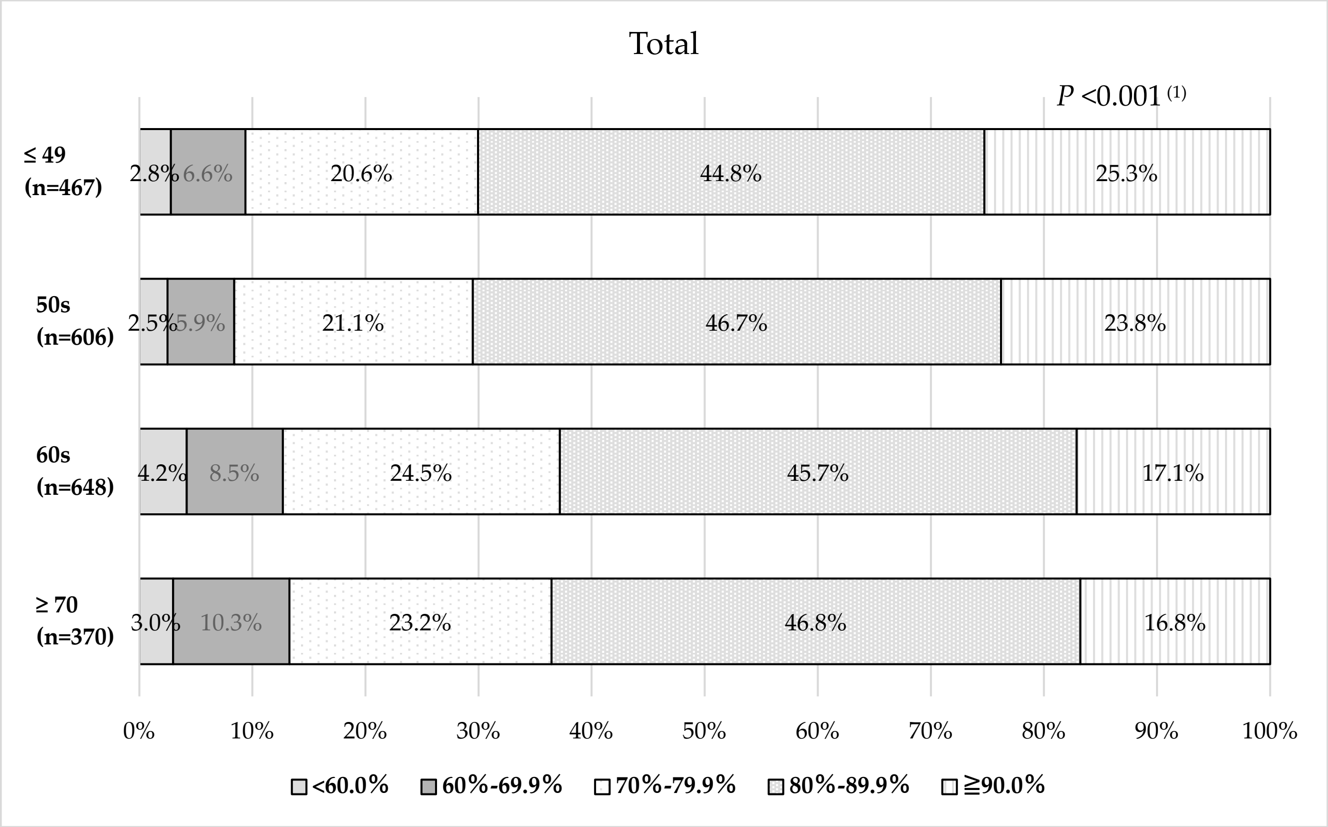

Figure S4A. Distribution of sleep efficiency by age groups.

A. All participants. B. Men. C. Women.

(1) Mantel–Haenszel test for trends.

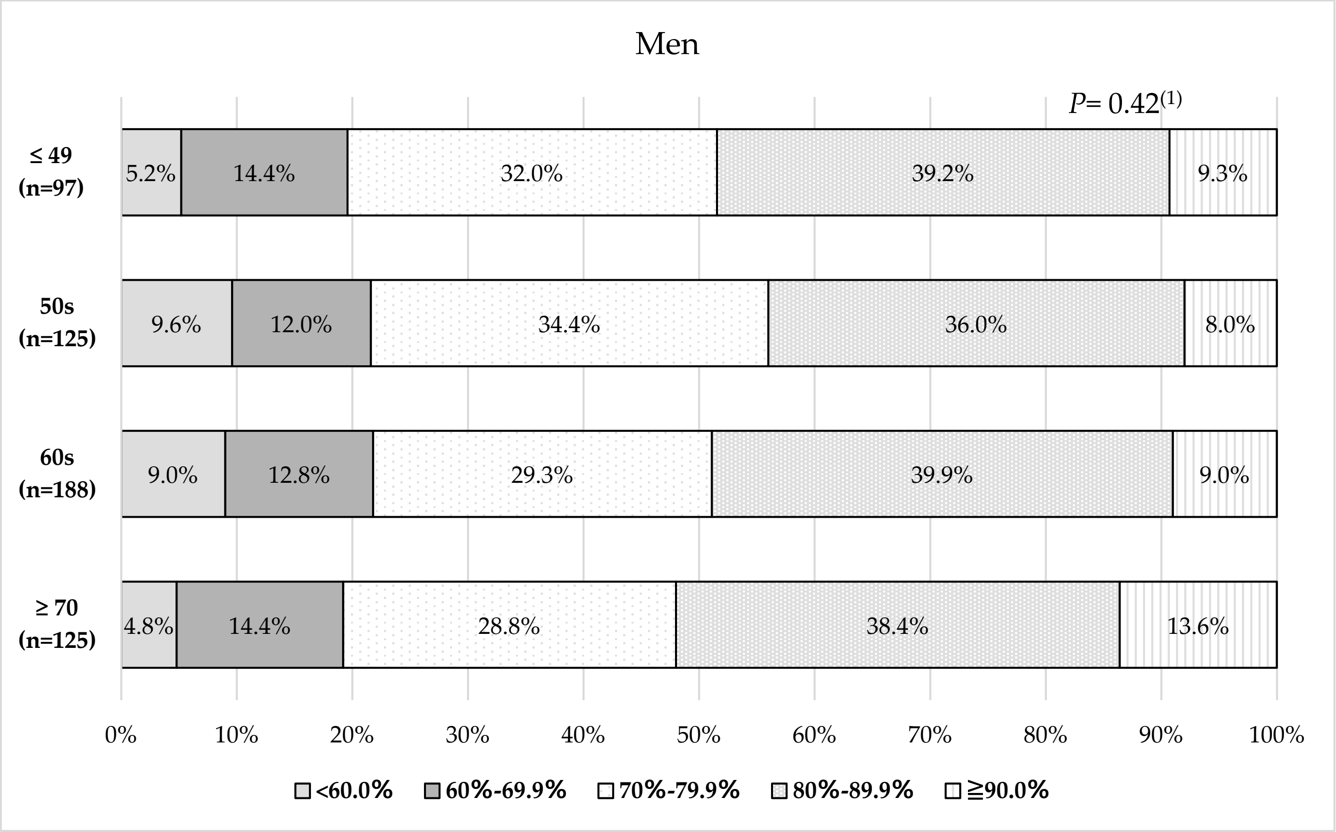

Figure S4B. Distribution of sleep efficiency by age groups

(1) Mantel–Haenszel test for trends.

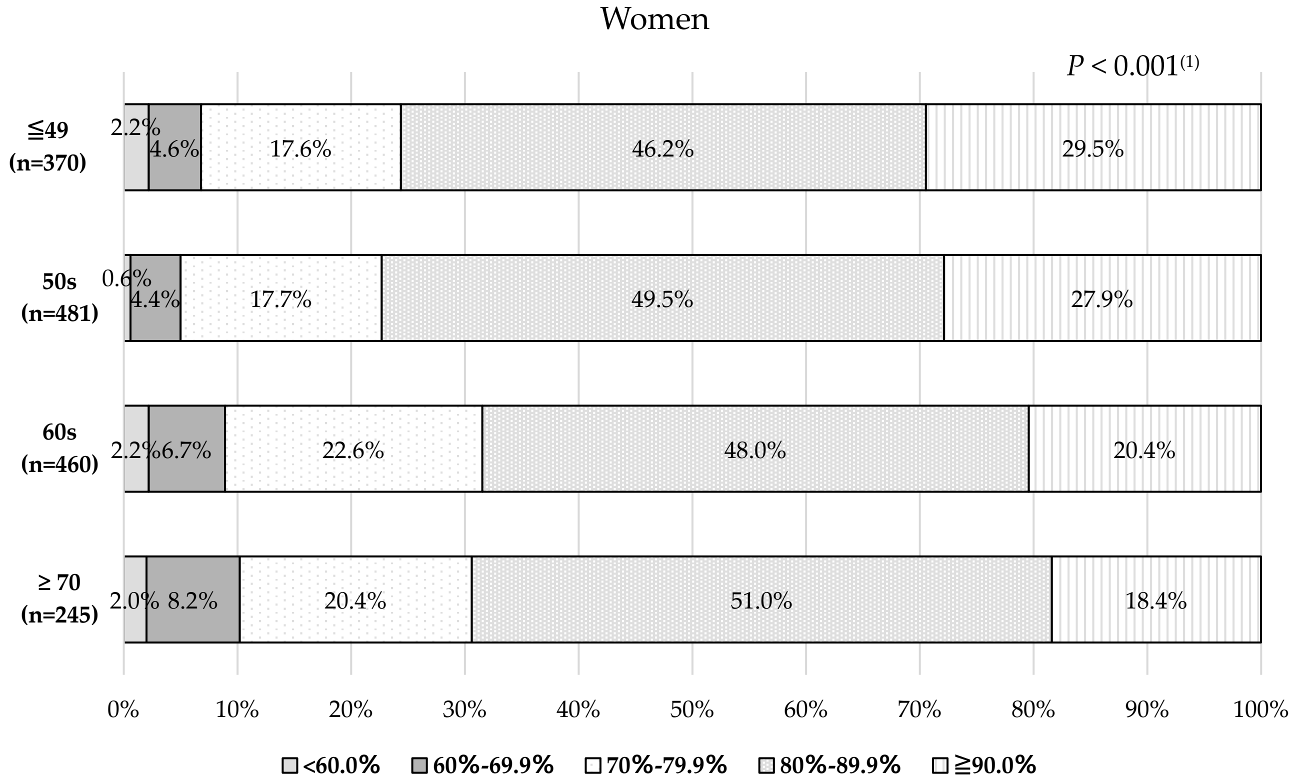

Figure S4C. Distribution of sleep efficiency by age groups

(1) Mantel–Haenszel test for trends.

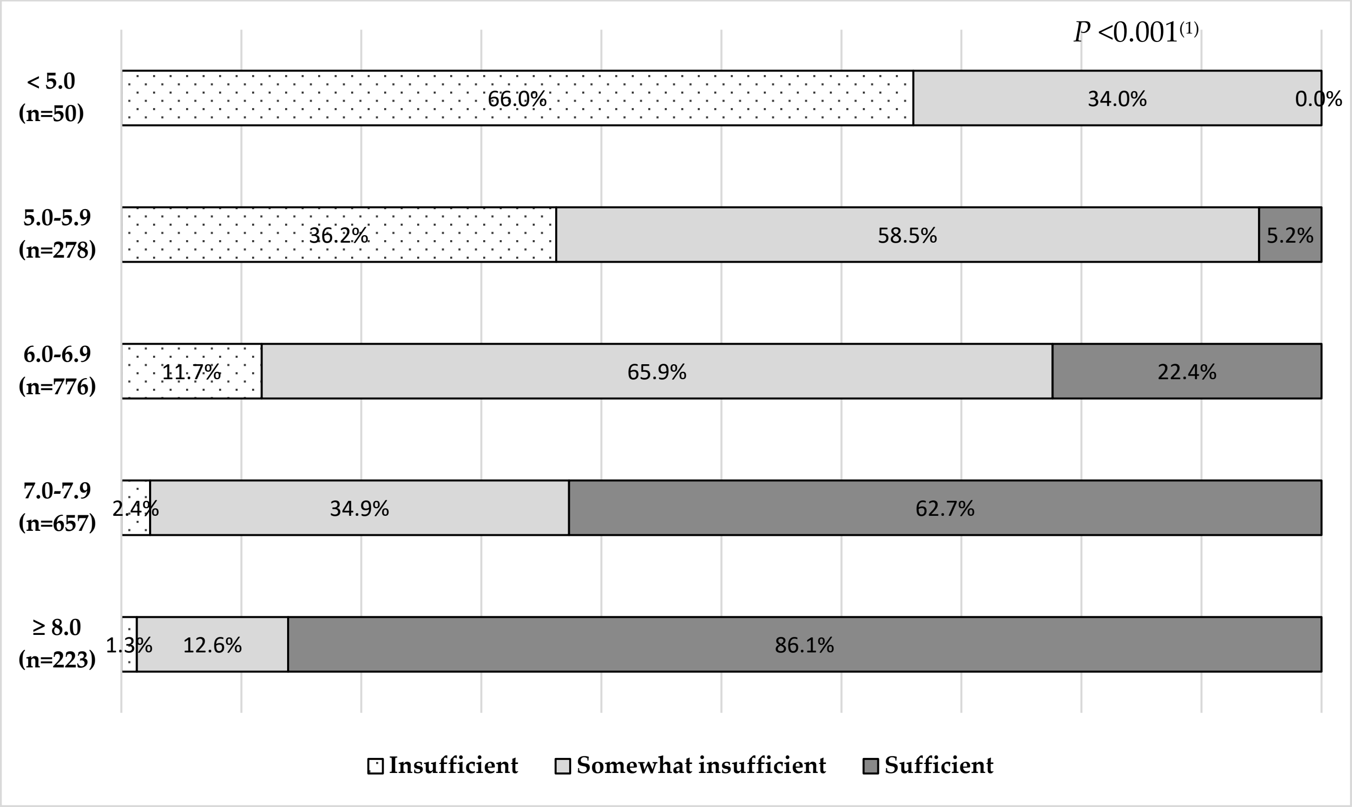

Figure S5. Self-rated sleep duration and perceived sleep insufficiency

(1) Mantel–Haenszel test for trends.
